# Comparative genomic analysis reveals varying levels of mammalian adaptation to coronavirus infections

**DOI:** 10.1371/journal.pcbi.1009560

**Published:** 2021-11-18

**Authors:** Sean B. King, Mona Singh

**Affiliations:** 1 Department of Molecular Biology, Princeton University, Princeton, New Jersey, United States of America; 2 Lewis-Sigler Institute for Integrative Genomics, Princeton University, Princeton, New Jersey, United States of America; 3 Department of Computer Science, Princeton University, Princeton, New Jersey, United States of America; National Center for Biotechnology Information (NCBI), UNITED STATES

## Abstract

Severe acute respiratory coronavirus 2 (SARS-CoV-2), the causative agent of COVID-19, is of zoonotic origin. Evolutionary analyses assessing whether coronaviruses similar to SARS-CoV-2 infected ancestral species of modern-day animal hosts could be useful in identifying additional reservoirs of potentially dangerous coronaviruses. We reasoned that if a clade of species has been repeatedly exposed to a virus, then their proteins relevant for viral entry may exhibit adaptations that affect host susceptibility or response. We perform comparative analyses across the mammalian phylogeny of angiotensin-converting enzyme 2 (ACE2), the cellular receptor for SARS-CoV-2, in order to uncover evidence for selection acting at its binding interface with the SARS-CoV-2 spike protein. We uncover that in rodents there is evidence for adaptive amino acid substitutions at positions comprising the ACE2-spike interaction interface, whereas the variation within ACE2 proteins in primates and some other mammalian clades is not consistent with evolutionary adaptations. We also analyze aminopeptidase N (APN), the receptor for the human coronavirus 229E, a virus that causes the common cold, and find evidence for adaptation in primates. Altogether, our results suggest that the rodent and primate lineages may have had ancient exposures to viruses similar to SARS-CoV-2 and HCoV-229E, respectively.

## Author summary

SARS-like coronaviruses, such as SARS-CoV and SARS-CoV-2, are a class of pathogens that have been a significant threat to human health. Across the animal kingdom, we see considerable differences in the severity of symptoms caused by these types of viruses. Previous research has shown adaptations to SARS-like viruses in known viral vectors such as Chinese Horseshoe bats. Uncovering evidence of adaptations to SARS-like viral infections in proteins of other hosts would shed light on whether additional mammalian clades have been previously exposed to these viruses. In our study, we characterize the evolution of the mammalian host receptor of SARS-like viruses, ACE2, across a broad mammalian phylogeny. Our comparative genomic insights reveal a pattern of rapid evolution of the ACE2/virus binding interface within the rodent clade, while finding little adaptation within primates or among other mammals not already known to be SARS hosts. These results suggest that some rodent species may have acquired some form of tolerance or resistance to infections from SARS-like coronaviruses, while non-adapted mammals and primates may be more susceptible to symptomatic disease.

## Introduction

Coronaviruses (CVs) are single-stranded RNA enveloped viruses that affect a wide range of mammals and birds, and cause contagious upper respiratory infections in humans [1]. Estimates of when CVs first appeared vary widely, from 300 million to 10,000 years ago [2,3]. CVs are capable of transmission between species and undergo adaptations to new host defenses [4]. There have been three recent zoonotic CV transmissions to humans that have had significant health impacts: severe acute respiratory coronavirus (SARS-CoV) in 2002 with ~8,000 infections and ~775 fatalities, Middle East respiratory syndrome (MERS-CoV) in 2012 with ~2,500 infections and ~850 fatalities, and most recently SARS-CoV-2, the cause of the COVID-19 pandemic [5,6]. While SARS-CoV, SARS-CoV-2, and MERS are likely of bat origin, the wide host range of CVs suggests there may be multiple other animal reservoirs of infectious CVs [7,8].

The spike proteins of SARS-CoV and SARS-CoV-2 bind ACE2 receptors to gain entrance to host cells [8]. Structural studies have identified the receptor binding domain within the SARS-CoV and SARS-CoV-2 spike proteins, and have demonstrated that the sites within the ACE2 protein that interact with SAR-CoV and SARS-CoV-2 are largely shared, with 17 of 20 sites overlapping [9,10,11,12,13]. Spike interaction sites within the ACE2 proteins of Chinese horseshoe bats, reservoirs for many viruses similar to SARS-CoV and SARS-CoV-2, have acquired adaptations that suggest bat genomes have gradually evolved in response to infections by these viruses [14,15,16,17]. The ACE2 receptors of several other species can be recognized by SARS-CoV-2, suggesting that SARS-CoV-2 may be able to infect a broad range of hosts [14,18]. Evolutionary analysis of the ACE2/spike protein interface across the mammalian phylogeny could help reveal additional taxa that have had as yet unidentified exposure, over evolutionary timescales, to SARS-like CVs (i.e., CVs that utilize ACE2 in a similar manner as SARS-CoV and SARS-CoV-2) and thus could potentially be reservoirs of SARS-like CVs.

Here, we perform a comparative analysis of ACE2 receptors across mammalian species as well as for spike proteins across SARS-like viruses, and compute for all positions in both sets of proteins evolutionary rates of amino acid substitutions as well as measures of positive selection. Overall, we find that sites within ACE2 and the spike protein that comprise the ACE2/spike protein interface evolve faster than other sites in these proteins, and are enriched amongst those that exhibit evidence of positive selection. Analysis of different mammalian clades reveals that there is minimal variation within ACE2 proteins across primates in spike-binding positions, whereas in rodents there is a significant enrichment for adaptive amino acid substitutions at positions comprising the ACE2-spike interaction interface. We also perform a comparative analysis of the APN receptor, which is used by HCoV-229E, a CV that interacts with the human APN receptor and is one agent responsible for the common cold [19]. In contrast to SARS-CoV and SARS-CoV-2, HCoV-229E has been widely circulating and infecting humans for a long time, and it has been hypothesized that an HCoV-229E-like ancestor may have been active for millions of years [2]. For the APN receptor, we find that sites which are critical for interactions with HCoV-229E have higher evolutionary rates than other sites in the receptor, and exhibit evidence for positive selection in the primate phylogeny. Our findings are consistent with the claim that rodents, but not primates, have been exposed to and adapted to SARS-like CVs over evolutionary time, whereas primates have been exposed to and adapted to HCoV-229E.

## Results

### The SARS-CoV-2 spike protein receptor binding domain shows evidence of adaptation

We first investigated the site-wise evolutionary rates of the 17 structurally determined ACE2 binding sites in the SARS-CoV-2 spike protein [9] (PDB:6M0J). Our set of 27 SARS-like spike proteins include proteins from SARS-CoV-2 and SARS-CoV and come from viruses that target known hosts including civets, bats, humans, and pangolins (S1 Table). For each position in the multiple sequence alignment of these proteins, we calculated the relative rate of evolution of amino acid substitutions using the Rate4Site program [20]. Rate4Site estimates the rate of evolution with a maximum likelihood method that considers the evolutionary tree relating the sequences; higher scores correspond to faster evolutionary rates and higher amino acid variability. For each position, we also calculated the Shannon entropy of the distribution of amino acids [21]; here a score of 0 corresponds to no variation and higher scores correspond to sites with increasing amino acid diversity. As compared to sites within the spike protein that do not bind ACE2, we find that ACE2 binding sites in the SARS-CoV-2 spike protein have both a significantly higher Shannon entropy, as well as a significantly higher mean relative evolutionary rate, as measured by Rate4Site (0.56 vs 0.17, P<0.001, and 1.89 vs -0.026, P<0.001, respectively; Mann Whitney U-test (MWU), S2 Table).

To investigate whether this increased rate of amino acid evolution corresponds to selective pressure on these binding sites, we next used phylogenetic methods to determine whether there are any binding sites exhibiting site-wide pervasive positive selection or episodic selection using the single-likelihood ancestor counting method (SLAC) and the mixed effects model of evolution (MEME), respectively [22,23,24] (see Materials and Methods). SLAC estimates the rates of non-synonymous (dN) and synonymous (dS) changes at each site, and we classified a site as exhibiting evidence for site-wide pervasive positive selection if its SLAC dN-dS value is greater than zero. MEME estimates positive selection for each site on each branch of the phylogenetic tree, and we classified a site as exhibiting evidence for episodic selection (i.e., positive selection occurring at any branch) if MEME’s reported Likelihood Ratio Test statistic is greater than one. Out of the 17 ACE2 binding sites within the SARS-CoV-2 spike protein, we find evidence for site-wide pervasive positive selection at seven sites and episodic selection at six sites, of which five overlap. Since pervasive positive selection and episodic selection are both difficult to detect at the level of individual sites and tend to rarely reach levels of statistical significance [22,23], we instead use a hypergeometric test to calculate whether sites found to have evidence of being under positive or episodic selection are found more frequently than expected amongst sites at the receptor binding interface. We find a 4.11 and 5.65-fold enrichment for site-wide pervasive positive and episodic selection, respectively, at binding sites for ACE2 versus the spike protein as a whole, meaning evidence for pervasive positive and episodic selection is found more frequently than expected among sites at the receptor binding interface (P<0.001 for both, hypergeometric (HG) test).

### ACE2 proteins across mammals exhibit accelerated evolution at the spike protein interface

**Fig 1:**
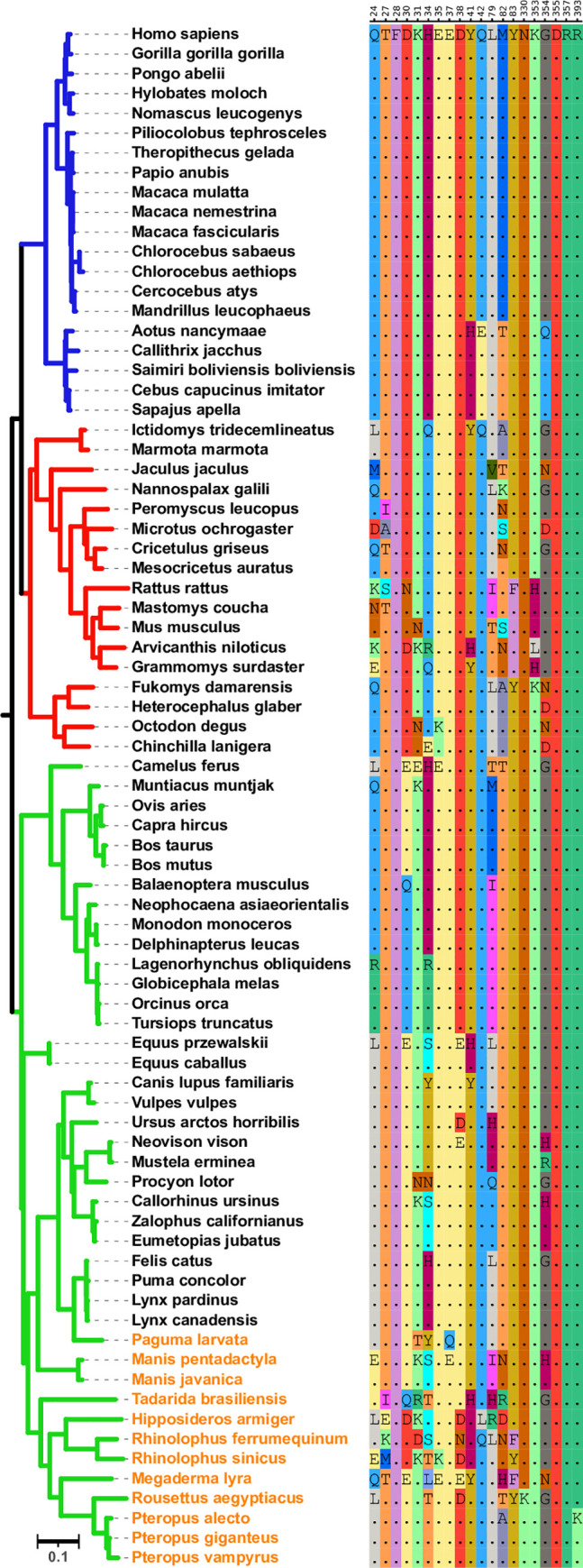
Phylogenetic tree of mammalian ACE2 sequences and residues that bind the viral spike protein. **(Left)** A maximum likelihood phylogenetic tree for the ACE2 receptors with primates, rodent, and other mammal lineages shown in blue, red, and green, respectively. Bats, civet, and pangolin are specified via orange text. Only one species per genus is shown. The tree was built using RAxML [26] and the visualization was created using the iTOL platform [27]. **(Right)** For each of the 20 sites within the human ACE2 sequence that binds the SARS-CoV-2 spike protein, the corresponding residues in the multiple sequence alignment for the other ACE2 sequences are shown. Numbering is according to amino acid position in the human ACE2 sequence. The bat and rodent species clades exhibit the most amino acid variation.

**Fig 2:**
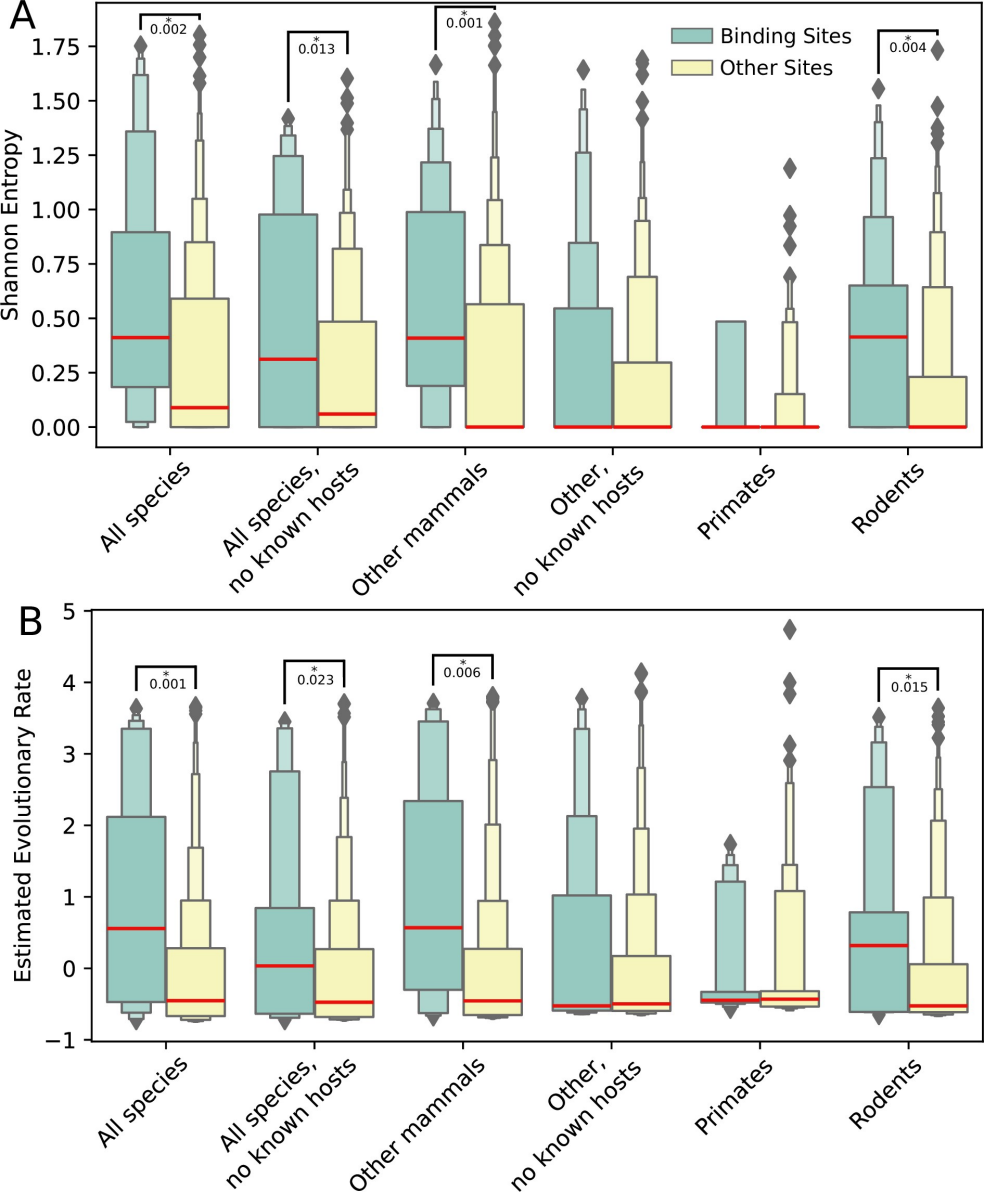
Variation and evolution across the ACE2 receptor at both the spike-binding and other surface-exposed extracellular regions. Letter-value (or boxen) plots of (A) Shannon entropy and (B) relative evolutionary rate estimates for sites within ACE2 that either bind (green) or do not bind (yellow) the SARS-CoV-2 spike protein, computed using sequences from each of the six species groups (all considered mammalian species, all mammalian species excluding bats, civets and pangolins—i.e., excluding several species that are known *Laurasiatheria* hosts of SARS-like viruses, along with additional bat species—mammalian species excluding primates and rodents, mammalian species excluding primates, rodents, bats, civets and pangolins, primates only, and rodents only). In letter-value plots, the widest boxes show half the data (from the 25th to 75th percentiles), while each successively narrower box shows half the remaining data. Median values are shown in red. For each measure and species group, comparisons between the binding sites and all other sites that yield P-values less than 0.05 (MWU) are shown. Spike-binding sites within the ACE2 receptor of the rodent, other mammals and “all species” groups have significantly more variation and faster rates of evolution than other surface-exposed extracellular sites, while primates and other mammals without known SARS hosts do not.

We next examined the host side of the ACE2-spike protein interface by analyzing the ACE2 protein across 78 non-redundant species in the broader mammalian phylogeny (Fig 1). We computed the Shannon entropies and Rate4Site-derived evolutionary rates computed for each site in the alignment of these sequences. Because spike-binding sites are on the surface of the extracellular domain of ACE2, in all the analyses that follow, we compared these values for spike-binding sites to other extracellular surface-exposed sites within ACE2, as the evolutionary pressure on other sites may be considerably different (e.g., buried residues tend to be conserved). The extracellular region of the structure spans amino acids 19–615, and to identify surface residues within this region, we used the ACE2 structure to find residues with any accessible surface area, as determined by PDBePISA [25]. We also performed our analysis on the full ACE2 sequence, which yielded very similar overall results (S3 and S4 Tables). We found that Shannon entropies and evolutionary rates are significantly higher for the 20 ACE2 sites that bind the SARS-CoV-2 spike protein [9] (PDB:6M0J) than those for other extracellular surface-exposed sites in these proteins (P = 0.002 and P = 0.001, respectively; MWU; Table 1 and Fig 2). We also analyzed these sites for evidence that this signal of accelerated evolution was due to selective pressure. Throughout the surface-exposed and extracellular portion of the ACE2 sequence, using the same criteria as described earlier, we identified ten binding sites with evidence of site-wide pervasive positive selection, as well as ten sites with evidence of episodic selection, seven of which overlap (S5 Table). Further, the number of sites with evidence for site-wide pervasive positive selection is 2.58-fold enriched within binding sites (P = 0.002; HG) and the number of sites with evidence for episodic selection is 2.71-fold enriched within binding sites (P = 0.001; HG).

**Table 1:** Mean evolutionary rates as determined by Shannon entropy (top) and Rate4Site (bottom) of ACE2 sites within each species group for spike-binding sites and all other surface-exposed extracellular sites.

**Shannon Entropy**						
	**All species**	**All species, no known hosts**	**Other mammals**	**Other mammals, no known hosts**	**Primates**	**Rodents**
Binding sites	0.617	0.490	0.590	0.314	0.097	0.434
Other sites	0.315	0.263	0.291	0.206	0.060	0.195
P-value	**0.002**	**0.013**	**0.001**	0.22	0.32	**0.004**
**Normalized Evolutionary Rates**						
	**All species**	**All species, no known hosts**	**Other mammals**	**Other mammals, no known hosts**	**Primates**	**Rodents**
Binding sites	0.959	0.610	1.01	0.367	0.0112	0.620
Other sites	-0.037	-0.024	-0.039	-0.014	-0.0004	-0.024
P-value	**0.001**	**0.023**	**0.0006**	0.30	0.36	**0.015**

The broader mammalian phylogeny we utilized includes bats, civets and pangolins—several of which are previously identified hosts of SARS-like viruses and have been shown to exhibit evidence of selection at the ACE2/spike binding interface [15,16,17]. We thus used these same measures of conservation and selection on a tree with these species excluded to identify whether this statistically significant signal could be entirely attributed to these already known hosts of SARS-like viruses. Surprisingly, even with sequences from these known hosts removed, we still find that, as compared to other surface-exposed extracellular sites, spike binding sites show significantly more amino acid variation and rapid evolution (P = 0.013 and P = 0.023, respectively; MWU; Table 1 and Fig 2), and evidence for both pervasive and episodic selection (2.21-fold enriched, P = 0.016, and 2.75-fold enriched, P = 0.004, respectively; HG). Given this accelerated evolutionary rate and considerable enrichment of sites with evidence for pervasive and episodic selection even with known SARS hosts removed, we hypothesized that there must be an additional set of species driving this selective signal outside of just the species already known to harbor SARS-like viruses.

### Mammalian subclades exhibit different evolutionary dynamics at the spike protein interface

To determine whether there is a particular clade that is dominating the signal of accelerated evolution across the broader mammalian phylogeny, we next looked at the three highest order clades in the phylogenetic tree of the ACE2 receptors: primates, rodents, and “other mammals” in the *Laurasiatheria* lineage.

In the primate sequence clade, which contains sequences from a diverse set of primates including great apes, New World monkeys, and Old World monkeys, we see very little difference in the evolutionary rates between the ACE2 spike-binding sites and other sites in the protein. Of the surface-exposed extracellular sites not involved in binding the spike protein, 83% are completely conserved across the primate phylogeny, while 16 of 20 total spike-binding sites (80%) are conserved across the entire clade. The four binding sites with any variation each appear to have a single amino acid change that originated in the common ancestor of the New World Monkeys. Furthermore, SARS-CoV-2 binding sites within the ACE2 receptors in primates are slightly under enriched for signals of site-wide pervasive positive or episodic selection (0.80-fold, P = 0.74, and 0.77-fold, P = 0.75, respectively; HG). Consistent with this, the Shannon entropies and estimated evolutionary rates of primate spike-binding sites are not significantly different from other surface-exposed extracellular sites (mean values of 0.097 vs 0.060, P = 0.32 and 0.0112 vs -0.0004, P = 0.36, respectively; MWU; Table 1 and Fig 2).

In the *Laurasiatheria* “other mammal” sequence clade, which together encompass a greater evolutionary distance than sequences from the primate clade, we see considerably more amino acid variation. Unlike the primate clade, the other mammals show a much more significant difference in evolutionary pattern between their spike binding and other sites (mean Shannon entropy values of 0.590 vs 0.291, P = 0.001 and mean estimated evolutionary rates of 1.01 vs -0.039, P = 0.006, respectively; MWU; Table 1). We also observe a significant enrichment of sites under site-wide pervasive positive or episodic selection within the binding sites (2.11-fold, P = 0.005, and 2.39-fold, P = 0.006, respectively; HG). While ACE2 sequences in this other mammal clade show significant patterns for adaptation at these sites, it is interesting to note that if we exclude ACE2 sequences from bats, civet and pangolin species (which include known SARS-hosts), the results change dramatically. This change brings the differences in Shannon entropies and evolutionary rates between binding and other sites to non-significance (means of 0.314 vs. 0.201, P = 0.22, and 0.367 vs. -0.014, P = 0.30, respectively; MWU; Table 1), and diminishes the signal for site-wide pervasive and episodic selection (1.69-fold, P = 0.10, and 1.53-fold, P = 0.26, respectively; HG). To contextualize this sharp change, we measured selection in just the nine bat species, comprised of 28 total sequences (S1 Table) which, as expected, show a strong signal of pervasive and episodic selection (4.02-fold, P<0.0001, 8.93-fold, P<0.0001, respectively; HG).

Like the “other mammal” clade, the rodent clade has greater overall amino acid variation in its ACE2 sequences when compared to primates. Further, we discover evidence for more substantial amino acid variation at the spike-binding interface as compared to the variation seen within the primate clade or the other mammal clade with known hosts of SARS-like viruses excluded (Shannon entropies of 0.434, 0.097, and 0.314, respectively) (Fig 2 and Table 1). Additionally, as compared to the total evolutionary rates of the other surface-exposed extracellular sites of the rodent ACE2 protein, the spike-binding sites have significantly higher sequence entropies and evolutionary rates (means of 0.434 vs. 0.195, P = 0.004, and 0.620 vs. -0.024, P = 0.015; MWU; Table 1 and Fig 2). Overall, these results indicate that the observed signal of accelerated evolution of these sites across the 78 species included in our mammalian phylogeny, when known hosts of SARS-like viruses are excluded, is largely being driven by the rodent clade.

We next explored the patterns of variation of spike-binding sites in the rodent ACE2 proteins by looking for signals of selective pressure at these sites. Within the total set of 20 spike-binding sites, we see evidence for site-wide pervasive positive selection at seven sites and episodic selection at five of these sites. These sites exhibit a respective 2.32 and 2.44-fold enrichment for selection as compared to other extracellular surface-exposed sites (P = 0.02 and P = 0.04, respectively; HG). Sites 24, 27, 30, 31, and 82 all show a signal of accelerated evolution as measured by their above-average evolutionary rates, as well as evidence for both site-wide pervasive and episodic selection. S1 Fig provides a representative illustration of this evolutionary pattern at site 24, showing repeated amino acid changes at internal nodes across the rodent phylogeny, which combined with quantitative evidence for selective pressure, suggests the possibility that repeated SARS-like infections in ancestral species may be a contributing factor to the evolution of these sites within the binding interface.

### Primate receptor adaptation to HCoV-229E-like CVs

While the lack of amino acid variation at the spike-binding interface within primate ACE2 proteins suggests that primates have not adapted to SARS-like infections, we next explored whether primate receptors for older CVs exhibited any evidence of adaption. The more ancient HCoV-229E CV is associated with the common cold and infected individuals typically exhibit relatively mild symptoms [28]. As HCoV-229E enters its host cells via interaction with the APN protein, we performed comparative analyses of the APN protein sequence across the primate clade. We note that comparative analysis of mammalian APN proteins has been performed previously [29]; however, that analysis was focused on the interaction interface between the porcine APN protein and two other CVs (transmissible gastroenteritis virus and porcine respiratory coronavirus), and the positions analyzed do not overlap the positions within APN that are involved in the interaction with HCoV-229E.

In contrast to the spike-binding sites within the ACE2 receptors, the Shannon entropies and evolutionary rates, computed per-site on the multiple sequence alignment of these receptor sequences, are significantly higher for the 10 APN sites that contact the HCoV-229E spike protein [30] (PDB:6U7E) than for its other sites (means of 0.294 vs 0.128, P = 0.024, and 0.68 vs -0.007, P = 0.036 respectively; MWU; S6 Table). Furthermore, three of these spike-binding sites have evidence for both site-wide pervasive positive and episodic selection. While sites with evidence of site-wide pervasive positive selection are not significantly enriched within binding sites (1.69-fold, P = 0.08; HG), those exhibiting episodic selection are far more strongly enriched within binding sites (3.43-fold, P = 0.008; HG). These results suggest that primate receptor binding domains may exhibit patterns of co-evolution with CVs when given sufficient evolutionary time.

## Discussion

CVs have a broad geographic distribution and have thrived in hosts across multiple clades of the tree of life, possibly for millions of years, though the timescale is a hotly debated topic [2]. We have shown evidence that suggests that mammalian clades have had varying levels of exposure to different CVs across an evolutionary timescale. We identified evidence for evolutionary adaptations as a response to CV infections at receptor binding interfaces in the ACE2 protein in rodents, while not observing this at the same sites in primates. While the evolution of this interface has been previously studied [14], our work characterizes rates of amino acid variation and evolution within ACE2 at positions comprising this interface, and further uncovers enrichment for pervasive and episodic selection within these positions. Additionally, we found evidence for adaptations in the primate APN protein at sites that interact with HCoV-229E.

In virus-host interactions, fast evolving viruses have the upper-hand. The ACE2 receptor binding domain of the SARS-CoV-2 spike protein is evolving very rapidly, posing a significant challenge for the more slowly evolving host to build defenses to this mechanism of invasion. Greater evolutionary timescales are required for animals to adapt to their viral hosts, and by examining the extent of these adaptations across clades, we can begin to provide evidence of the age of particular virus-host interactions and this may reveal the susceptibility to CV infections or disease of various animal hosts. The potential for severe symptoms associated with SARS-CoV-2 infections in humans, when contrasted with the apparent lack of symptoms in bat species that are known hosts of SARS-like viruses, suggests that these bats may have been interacting with similar CVs for a longer period, though significant differences in immune function between these bats and humans also contribute to this symptomatic discrepancy [14,16,17,31,32]. Overall, our results suggest that not only are SARS-like infections novel to modern humans, but there exists little evidence indicating that ACE2-binding CVs have been a significant evolutionary driver throughout the entire primate phylogeny.

The human CV NL63 (HCoV-NL63), which causes mild to moderate respiratory symptoms but is associated with more severe illness in very young, elderly, and immune-compromised individuals, also enters host cells by binding the ACE2 receptor. Overall, NL63-binding sites within ACE2 exhibit a similar pattern of very minimal variation across the primate phylogeny, suggesting little adaptation to this particular infection (S2 Fig). While NL63 has been hypothesized to be circulating within humans for longer than SARS-CoV or SARS-Cov-2 [33], the lack of adaptation further suggests that either NL63-like CVs have not been circulating within primates on an evolutionary timescale, or that it has always been a comparatively mild infection and does not warrant the possible fitness cost of ACE2 adaptations.

In contrast to the other mammalian clades we considered, our data suggest that rodents may have been exposed to repeated SARS-like CV infections for a considerable evolutionary period. Notably, for all five sites where we see a significant signal of both selection and rapid evolution in rodents (sites 24, 27, 30, 31 and 82), deep mutational scanning has revealed that they are critical for spike binding, with several substitutions at each of them strongly affecting ACE2-spike binding affinities [34]. Evidence for positive selection at ACE2 binding sites within rodents show an evolutionary pattern similar to bats, suggesting that these adaptations may confer either some level of tolerance of SARS-like infections (e.g., infections with mild symptoms) or complete resistance to cellular entry of SARS-like viruses due to the inability of their spike proteins to bind rodent ACE2. Mice infected with SARS-CoV and SARS-CoV-2 have been found to have subclinical or very mild disease [35,36,37], lending support to rodents’ tolerating—as opposed to being resistant to—these infections. Additionally, two other CVs that infect humans, HKU1 and HCoV-OC43, are thought to have originated in rodents [1]. Nevertheless, rodents are not currently known to be a reservoir of SARS-like viruses. Further, while it remains unknown whether SARS-like viruses would be able to be robustly transmitted from rodents to humans, this possibility warrants further study.

While our evolutionary analysis suggests that the primate interaction with ACE2-binding CVs like SARS-CoV-2 is very recent, we have shown that more ancient primate-CV interactions may have driven defensive adaptations. Evidence for selection at the primate APN-HCoV-229E interface suggests that similar CV infections may have been circulating on an evolutionary timescale, and if this is true, it also provides evidence that primates can exhibit an evolutionary response as a CV host when given a sufficient period of time. While defensive adaptations over evolutionary distances do not have a direct impact on the human health response to a novel infection, successful adaptations to other CVs suggests that population-level variation may be a contributing factor to differing health outcomes in human hosts. Indeed, it may be possible to detect potential future adaptations by collecting variant data and associating them to favorable SARS-CoV-2 patient outcomes.

Our work sheds light on ancestral mammalian relationships with coronaviruses by analyzing ACE2 and APN, but there are other host proteins that are known to mediate CV infections. DPP4, CEACAM1, and non-primate APN/CD13 all also serve as points of entry for various CVs [38], and characterizing the adaptation, or lack thereof, in these proteins would serve as the natural continuation of our work and may reveal other host-virus relationships.

While several species of bats are currently perceived as the most consequential animal host of SARS-CoV-2, our data suggest that other animals, either clades or several species within a clade, may have had ancestral exposures to SARS-like viruses that have resulted in evolutionary adaptations at the ACE2-spike interface that may confer some form of tolerance or resistance to infections from SARS-like viruses. Additionally, we have found little adaptation in primates and many other mammalian species and thus these species may be susceptible to SARS-like viruses. Applications of zoonotic transmission mitigation measures in susceptible hosts have already been put into action, perhaps most notably, the Danish government’s order to cull its entire farmed mink population. Continuing to explore adaptations in CV animal hosts will be important both for understanding which animals have possibly adapted tolerance mechanisms through ancient exposures and may be carriers of SARS-like viruses, as well as for finding those that are vulnerable symptomatic hosts, as humans have unfortunately proven to be.

## Materials and methods

### Constructing host receptor and viral spike orthogroup datasets

We used six targeted BLAST searches [39] to construct our sequence datasets (see S7 Table for the query accessions and taxa excluded from the searches). To build our datasets of viral spike proteins, primate ACE2 proteins and rodent ACE2 proteins, we used as queries the SARS-CoV-2 spike protein, human ACE2 protein and mouse ACE2 protein, respectively. To build our dataset of other mammal ACE2 proteins, we used as queries the human ACE2 protein and, to ensure coverage of bat ACE2 sequences, the *Rhinolophus sinicus* ACE2 sequence; for these searches, the primate and rodent taxa were excluded.

For each BLAST search, we extracted the top 50 BLAST hits. We chose to limit our BLAST searches to the first 50 sequences because a smaller number of sequences reduced overall coverage of species, while a larger number of sequences resulted in overlapping hits between the different BLAST queries. We removed sequences from genomes that are not assembled at either the completed scaffold or chromosome level; that is, since our analysis is focused on amino acid variation, we exclude sequences that are more likely to include errors due to sequencing or assembly issues. We excluded sequences explicitly annotated as partial or low quality for similar reasons. We removed duplicate identical sequences to obtain non-redundant datasets. When multiple isoforms were retrieved within a single species, we selected the isoform with the highest sequence identity to the query.

Altogether, we assembled a dataset of 27 homologs of the spike glycoprotein of SARS-CoV-2; three datasets of ACE2 receptors from primates, rodents, and “other mammals” from the *Laurasiatheria* lineage, consisting of 20, 17, and 41 sequences respectively; and a dataset of primate orthologs of the human APN protein, consisting of 19 sequences. Accession numbers for all sequences in our final datasets are given in S1 Table.

For each group of sequences, we used MAFFT version 7.390 with default parameters to build a multiple sequence alignment [40]. Columns in the alignments containing sites within 4Å of their respective ligands were denoted as “binding sites” [9,29] (PDB:6M0J, 6U7E). There are 20 spike-binding sites within the ACE2 proteins, 17 ACE2-binding sites within the spike protein, and 10 spike-binding sites within the APN protein. We identified solvent-exposed residues in the ACE2 sequences using PDBePISA [25], which defines surface residues as those with an accessible surface area of greater than 0Å^2^; we note that the accessible surface areas for spike-binding residues vary from 12.9Å^2^ to 135.6Å^2^. The extracellular region in the human ACE2 protein spans amino acids 19–615 and PDBePISA identified 536 solvent-exposed amino acids within this region.

### Measuring the rate of evolution of amino acid sites in CV/receptor interfaces

We used the maximum likelihood phylogenetic inference tool RAxML with the PROTGAMMABLOSUM62 substitution matrix [26] to build maximum likelihood trees of the ACE2 proteins for each of the three species groups separately, of all the ACE2 proteins across these groups, of the APN proteins, and of the viral spike proteins. Using these trees as guides, for each site we calculated the z-score normalized maximum likelihood estimate of the relative rate of evolution of amino acid substitution using the Rate4Site program with the default parameters [20]. For each position within the multiple sequence alignment, we also calculated the Shannon entropy to measure amino acid variation [21]. For the SARS-CoV-2 spike, ACE2, and APN proteins, we calculated whether binding sites are evolving significantly more rapidly than other sites by comparing the maximum likelihood evolutionary rate and Shannon entropy measures between binding sites and all other sites using a MWU test.

### Identifying sites exhibiting evidence of selective pressure

We measured codon-level selective pressure acting at the spike/ACE2 and spike/APN binding interfaces by acquiring from the NCBI the corresponding nucleotide sequences for the proteins included in the previous analyses. We performed a total codon alignment of the nucleotide sequences with the pal2nal program (with default parameters), using the aligned amino acid sequences as input to guide the codon-based alignment of the corresponding nucleotide sequences [41,42]. We calculated branch length adjusted dN-dS values and a sitewise episodic selection measure at each codon in the multiple sequence alignment using, respectively, the single-likelihood ancestor counting method and the mixed effects model of evolution from the datamonkey software suite [22,23,24]. The dN-dS values are larger positive values for a site when most lineages exhibit positive selection whereas episodic selection measures aim to identify whether at least one lineage exhibits positive selection. For each site, we classified it as exhibiting evidence for site-wide pervasive positive selection if its SLAC dN-dS value is greater than zero, and exhibiting evidence for episodic selection if MEME’s reported Likelihood Ratio Test statistic is greater than one. Since site-wide pervasive positive and episodic selection are difficult to detect at the level of individual sites and are in general rarely found to reach levels of statistical significance [22,23], we instead use a hypergeometric test to calculate whether sites found to have evidence of being under positive or episodic selection are found more frequently than expected amongst sites at the receptor binding interface.

## Supporting information

S1 Fig Site 24 of the rodent ACE2 receptor illustrates a pattern of positive selection. Rodent site 24 has multiple non-synonymous changes occurring both at internal nodes and terminal leaves. The corresponding amino acids are displayed on each node and each leaf, and the *Homo sapiens* reference codon is also included.
(TIF)

S2 Fig Multiple sequence alignment of primate NL63 binding sites. The 16 ACE2 sites located at the NL63 spike/ACE2 interface are shown across the 20 primate species used in the analysis.
(TIF)

S1 Table Protein accession numbers, names, and common species names for all sequences. Common names are provided for all non-viral species. In the case of species with multiple sequences, sequences used in Fig 1 are underlined, and correspond to those that are most similar to the human sequence.
(XLSX)

S2 Table Variation and positive selection measures of sites at the viral spike protein ACE2/SARS-CoV-2 binding interface. The 17 sites in the SARS-CoV-2 spike protein that are involved in binding the ACE2 receptor are shown. For each site, measures of normalized evolutionary rate by Rate4Site [20], amino acid variation using Shannon entropy [21], branch-scaled dN-dS from SLAC [22], and likelihood ratio test (LRT) from MEME [23] are included.
(XLSX)

S3 Table Mean evolutionary rates as determined by Shannon entropy (top) and Rate4Site (bottom) of ACE2 sites within each species group for spike-binding sites and all other sites. The same evolutionary rate calculations as Table 1 when sites within ACE2 are not restricted to surface, extracellular sites.
(XLSX)

S4 Table Enrichment of pervasive and episodic selection within each species group. The percent of sites with evidence for pervasive and episodic selection are reported for all ACE2 sites and spike binding sites when sites within ACE2 are not restricted to surface, extracellular sites. The fold enrichment for selected sites at the binding interface is shown, as well as the hypergeometric P value for significant enrichment.
(XLSX)

S5 Table Variation and positive selection measures of sites at the mammalian ACE2/SARS-CoV-2 binding interface. The 20 sites in the ACE2 receptor that are involved in binding the SARS-CoV-2 spike protein are shown. For each site, measures of normalized evolutionary rate by Rate4Site [20], amino acid variation using Shannon entropy [21], branch-scaled dN-dS from SLAC [22], and likelihood ratio test (LRT) from MEME [23] are included. The measures are shown when computed using all mammalian sequences, as well as when using sequences from each of the six species groups (all considered mammalian species, all mammalian species excluding known hosts of SARS-like viruses, mammalian species excluding primates and rodents, mammalian species excluding primates, rodents, and known hosts of SARS-like viruses, primates only, and rodents only).
(XLSX)

S6 Table Variation and positive selection measures of sites at the primate APN/HCoV-229E binding interface. The 10 sites in the APN receptor that are involved in binding the HCoV-229E spike protein are shown. For each site, measures of normalized evolutionary rate by Rate4Site [20], amino acid variation using Shannon entropy [21], branch-scaled dN-dS from SLAC [22], and likelihood ratio test (LRT) from MEME [23] are included. Site 292 dN-dS and LRT values required the removal of one species from the alignment that contains a gap at this site.
(XLSX)

S7 Table Protein accession numbers and excluded taxonomy IDs associated with each BLAST query. The SARS-CoV-2 sequence was used to build the protein alignment measuring variation across related SARS-like viruses. The human ACE2 sequence was used to query sequences for the primate and other mammal alignments, *Rhinolophus sinicus* was used to query bat sequences to add to the other mammals alignment, and the *Mus musculus* sequence was used to query sequences for the rodent alignment.
(XLSX)

## References

[1] Cui J, Li F, Shi ZL. Origin and evolution of pathogenic coronaviruses. Nat Rev Microbiol. 2019;17(3):181–92. doi: 10.1038/s41579-018-0118-9 30531947 PMC7097006

[2] Wertheim JO, Chu DKW, Peiris JSM, Pond SL, Poon LLM. A Case for the Ancient Origin of Coronaviruses. J Virol. 2013;87(12):7039–45. doi: 10.1128/JVI.03273-12 23596293 PMC3676139

[3] Woo PCY, Lau SKP, Huang Y, Yuen KY. Coronavirus diversity, phylogeny and interspecies jumping. Exp Biol Med. 2009;234(10):1117–27. doi: 10.3181/0903-MR-94 19546349

[4] Wu K, Peng G, Wilken M, Geraghty RJ, Li F. Mechanisms of host receptor adaptation by severe acute respiratory syndrome coronavirus. J Biol Chem. 2012;287(12):8904–11. doi: 10.1074/jbc.M111.325803 22291007 PMC3308800

[5] De Wit E, Van Doremalen N, Falzarano D, Munster VJ. SARS and MERS: Recent insights into emerging coronaviruses. Nat Rev Microbiol. 2016;14(8):523–34. doi: 10.1038/nrmicro.2016.81 27344959 PMC7097822

[6] World Health Organization. Weekly Epidemiological Update on COVID-19. 2020;(October).

[7] Tazerji S, Magalhães Duarte P, Rahimi P, Shahabinejad F, Dhakal S, Singh Malik Y, et al. Transmission of severe acute respiratory syndrome coronavirus 2 (SARS-CoV-2) to animals: An updated review. J Transl Med. 2020;18(1):1–11. doi: 10.1186/s12967-019-02189-8 32957995 PMC7503431

[8] Zhou P, Yang X-L, Wang XG, Hu B, Zhang L, Zhang W, et al. A pneumonia outbreak associated with a new coronavirus of probable bat origin. Nature. 2020;579(7798):270–3. doi: 10.1038/s41586-020-2012-7 32015507 PMC7095418

[9] Lan J, Ge J, Yu J, Shan S, Zhou H, Fan S, et al. Structure of the SARS-CoV-2 spike receptor-binding domain bound to the ACE2 receptor. Nature. 2020;581(7807):215–20. doi: 10.1038/s41586-020-2180-5 32225176

[10] Li F, Li W, Farzan M, Harrison SC. Structural biology: Structure of SARS coronavirus spike receptor-binding domain complexed with receptor. Science (80). 2005;309(5742):1864–8. 10.1126/science.1116480 16166518

[11] Shang J, Ye G, Shi K, Wan Y, Luo C, Aihara H, et al. Structural basis of receptor recognition by SARS-CoV-2. Nature. 2020;581(7807):221–4. doi: 10.1038/s41586-020-2179-y 32225175 PMC7328981

[12] Wang Q, Zhang Y, Wu L, Niu S, Song C, Zhang Z, et al. Structural and Functional Basis of SARS-CoV-2 Entry by Using Human ACE2. Cell. 2020;181(4):894–904.e9. doi: 10.1016/j.cell.2020.03.045 32275855 PMC7144619

[13] Yan R, Zhang Y, Li Y, Xia L, Guo Y, Zhou Q. Structural basis for the recognition of SARS-CoV-2 by full-length human ACE2. Science (80). 2020;367(6485):1444–8. doi: 10.1126/science.abb2762 32132184 PMC7164635

[14] Damas J, Hughes GM, Keough KC, Painter CA, Persky NS, Corbo M, et al. Broad host range of SARS-CoV-2 predicted by comparative and structural analysis of ACE2 in vertebrates. Proc Natl Acad Sci U S A. 2020;117(36):22311–22. doi: 10.1073/pnas.2010146117 32826334 PMC7486773

[15] Guo H, Hu B-J, Yang X-L, Zeng L-P, Li B, Ouyang S, et al. Evolutionary Arms Race between Virus and Host Drives Genetic Diversity in Bat Severe Acute Respiratory Syndrome-Related Coronavirus Spike Genes. J Virol. 2020;94(20):1–15. doi: 10.1128/JVI.00902-20 32699095 PMC7527062

[16] Frank H, Enard D, Boyd S. Exceptional diversity and selection pressure on SARS-CoV and SARS-CoV-2 host receptor in bats compared to other mammals. bioRxiv. 2020;1–22. doi: 10.1101/2020.04.20.051656 35892217 PMC9326293

[17] Demogines A, Farzan M, Sawyer SL. Evidence for ACE2-Utilizing Coronaviruses (CoVs) Related to Severe Acute Respiratory Syndrome CoV in Bats. J Virol. 2012;86(11):6350–3. doi: 10.1128/JVI.00311-12 22438550 PMC3372174

[18] Hu B, Guo H, Zhou P, Shi ZL. Characteristics of SARS-CoV-2 and COVID-19. Nat Rev Microbiol. 2020;(December). 10.1038/s41579-020-00459-7 PMC7537588 33024307

[19] Kolb AF, Hegyi A, Siddell SG. Identification of residues critical for the human coronavirus 229E receptor function of human aminopeptidase N. J Gen Virol. 1997;78(11):2795–802. doi: 10.1099/0022-1317-78-11-2795 9367365

[20] Pupko T, Bell RE, Mayrose I, Glaser F, Ben-Tal N. Rate4Site: An algorithmic tool for the identification of functional regions in proteins by surface mapping of evolutionary determinants within their homologues. Bioinformatics. 2002;18:71–7. doi: 10.1093/bioinformatics/18.suppl_1.s71 12169533

[21] Capra JA, Singh M. Predicting functionally important residues from sequence conservation. Bioinformatics. 2007;23(15):1875–82. doi: 10.1093/bioinformatics/btm270 17519246

[22] Pond SL, Frost SDW. Not so different after all: A comparison of methods for detecting amino acid sites under selection. Mol Biol Evol. 2005;22(5):1208–22. doi: 10.1093/molbev/msi105 15703242

[23] Murrell B, Wertheim JO, Moola S, Weighill T, Scheffler K, Pond SL. Detecting individual sites subject to episodic diversifying selection. PLoS Genet. 2012;8(7). doi: 10.1371/journal.pgen.1002764 22807683 PMC3395634

[24] Weaver S, Shank SD, Spielman SJ, Li M, Muse S V., Pond SL. Datamonkey 2.0: A modern web application for characterizing selective and other evolutionary processes. Mol Biol Evol. 2018;35(3):773–7. doi: 10.1093/molbev/msx335 29301006 PMC5850112

[25] Krissinel E, Henrick K. Inference of Macromolecular Assemblies from Crystalline State. J Mol Biol. 2007;372(3):774–97. doi: 10.1016/j.jmb.2007.05.022 17681537

[26] Stamatakis A. RAxML version 8: A tool for phylogenetic analysis and post-analysis of large phylogenies. Bioinformatics. 2014;30(9):1312–3. doi: 10.1093/bioinformatics/btu033 24451623 PMC3998144

[27] Letunic I, Bork P. Interactive Tree of Life (iTOL) v4: Recent updates and new developments. Nucleic Acids Res. 2019;47(W1):256–9. doi: 10.1093/nar/gkz239 30931475 PMC6602468

[28] Dijkman R, van der Hoek L. Human coronaviruses 229E and NL63: Close yet still so far. J Formos Med Assoc. 2009;108(4):270–9. doi: 10.1016/S0929-6646(09)60066-8 19369173 PMC7135404

[29] Enard D, Cai L, Gwennap C, Petrov D. Viruses are a dominant driver of protein adaptation in mammals. eLife. 2016;5:e12469. doi: 10.7554/eLife.12469 27187613 PMC4869911

[30] Li Z, Tomlinson ACA, Wong AHM, Zhou D, Desforges M, Talbot PJ, et al. The human coronavirus HCoV-229E S-protein structure and receptor binding. elife. 2019;8:1–22. doi: 10.7554/eLife.51230 31650956 PMC6970540

[31] Banerjee A, Baker ML, Kulcsar K, Misra V, Plowright R, Mossman K. Novel Insights Into Immune Systems of Bats. Front Immunol. 2020;11(January):1–15. doi: 10.3389/fimmu.2020.00001 32117225 PMC7025585

[32] Zhang SY, Cui J, Han NIJ, Streicker D, Li G, Tang XC, et al. Evolutionary relationships between bat coronaviruses and their hosts. Emerg Infect Dis. 2007;13(10):1526–32. doi: 10.3201/eid1310.070448 18258002 PMC2851503

[33] Pyrc K, Dijkman R, Deng L, Jebbink MF, Ross HA, Berkhout B, et al. Mosaic Structure of Human Coronavirus NL63, One Thousand Years of Evolution. J Mol Biol. 2006;364(5):964–73. doi: 10.1016/j.jmb.2006.09.074 17054987 PMC7094706

[34] Chan KK, Dorosky D, Sharma P, Abbasi SA, Dye JM, Kranz DM, et al. Engineering human ACE2 to optimize binding to the spike protein of SARS coronavirus 2. Science (80). 2020;369(6508):1261–5. doi: 10.1126/science.abc0870 32753553 PMC7574912

[35] Fagre A et al. SARS-CoV-2 infection, neuropathogenesis and transmission among deer mice: Implications for reverse zoonosis to New World rodents. BioRxiv. 2020. doi: 10.1101/2020.08.07.241810 34010360 PMC8168874

[36] Wentworth DE, Gillim-Ross L, Espina N, Bernard KA. Mice susceptible to SARS coronavirus. Emerg Infect Dis. 2004;10(7):1293–6. doi: 10.3201/eid1007.031119 15324552 PMC3323317

[37] Montagutelli X, Prot M, Levillayer L, Salazar EB, Jouvion G, Conquet L, et al. The B1.351 and P.1 variants extend SARS-CoV-2 host range to mice. bioRxiv. 2021;2:1–16.

[38] Millet JK, Jaimes JA, Whittaker GR. Molecular diversity of coronavirus host cell entry receptors. FEMS Microbiol Rev. 2020;14(October 2020):1–16. 10.1093/femsre/fuaa057 PMC7665467 33118022

[39] Altschul SF, Gish W, Miller W, Myers EW, Lipman DJ. Basic local alignment search tool. J Mol Biol. 1990;215(3):403–10. doi: 10.1016/S0022-2836(05)80360-2 2231712

[40] Katoh K, Standley DM. MAFFT multiple sequence alignment software version 7: Improvements in performance and usability. Mol Biol Evol. 2013;30(4):772–80. doi: 10.1093/molbev/mst010 23329690 PMC3603318

[41] Sievers F, Higgins DG. Clustal Omega for making accurate alignments of many protein sequences. Protein Sci. 2018;27(1):135–45. doi: 10.1002/pro.3290 28884485 PMC5734385

[42] Suyama M, Torrents D, Bork P. PAL2NAL: Robust conversion of protein sequence alignments into the corresponding codon alignments. Nucleic Acids Res. 2006;34:609–12. doi: 10.1093/nar/gkl315 16845082 PMC1538804

