# Supplementary figures and images for: Comparative genomic analysis reveals varying levels of mammalian adaptation to coronavirus infections
Source: PLoS Comput Biol. 2021 Nov 18;17(11):e1009560. doi: 10.1371/journal.pcbi.1009560 (PMC8601562; doi:10.1371/journal.pcbi.1009560)

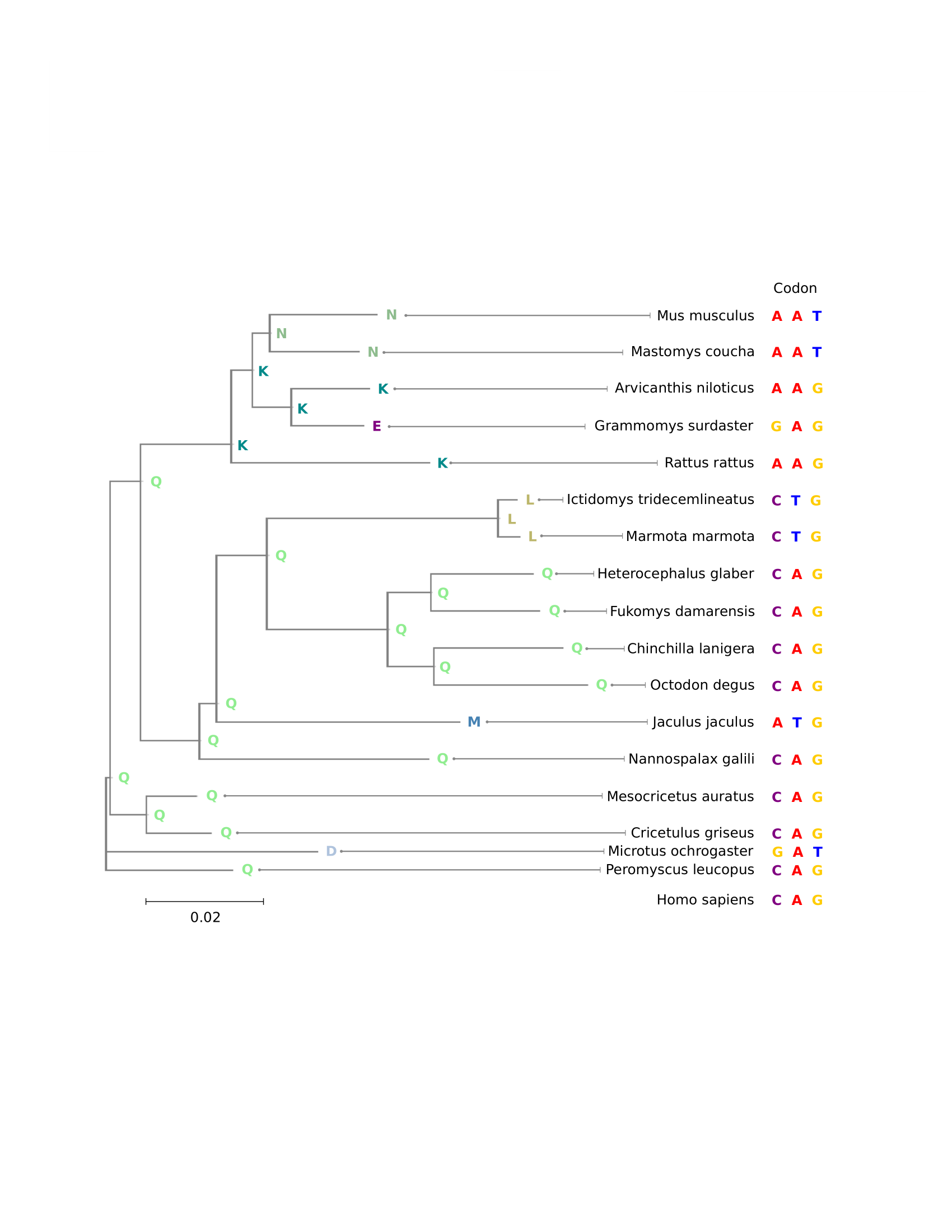

Supplement: S1 Fig — Rodent site 24 has multiple non-synonymous changes occurring both at internal nodes and terminal leaves. The corresponding amino acids are displayed on each node and each leaf, and the Homo sapiens reference codon is also included. (TIF) [file pcbi.1009560.s001.tif]

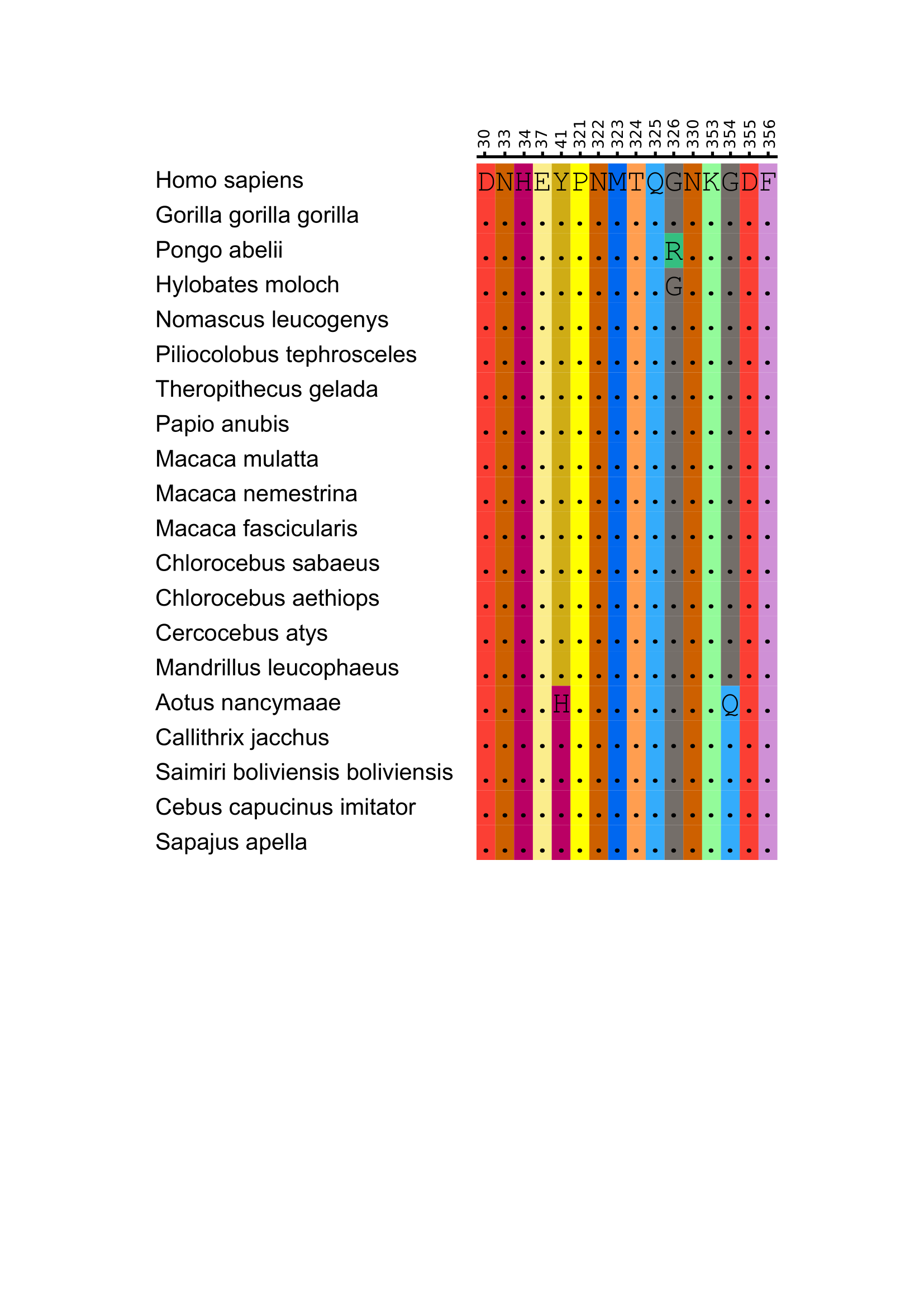

Supplement: S2 Fig — The 16 ACE2 sites located at the NL63 spike/ACE2 interface are shown across the 20 primate species used in the analysis. (TIF) [file pcbi.1009560.s002.tif]
